# Validation of Blubber Progesterone Concentrations for Pregnancy Determination in Three Dolphin Species and a Porpoise

**DOI:** 10.1371/journal.pone.0069709

**Published:** 2013-07-30

**Authors:** Marisa L. Trego, Nicholas M. Kellar, Kerri Danil

**Affiliations:** Southwest Fisheries Science Center, National Marine Fisheries Service, National Oceanic & Atmospheric Administration, La Jolla, California, United States of America; CNRS, France

## Abstract

Recent studies have validated the use of biopsies as a minimally invasive way to identify pregnant females in several species of wild cetaceans: *Balaenaptera* *acutorostrata* *,* *Delphinus* *delphis* *,* *Lissodelphis* *borealis* , and *Lagenorhynchus* *obliquidens* . These studies found that progesterone (P4) concentrations quantified from blubber attached to biopsy samples is diagnostic of pregnancy. Here we examine a broader group of cetacean species in efforts to investigate how progesterone levels vary between species with respect to pregnancy status. We compared P4 concentrations in blubber collected from fishery bycatch and beach-stranded specimens for 40 females of known reproductive condition from *Delphinus* *capensis* (n = 18), *Stenella* *attenuata* (n = 8), *S* *. longirostris* (n = 6), and *Phocoenoides* *dalli* (n = 8). The P4 concentrations were different (t = -7.1, *p* = 1.79E-08) between pregnant and non-pregnant animals in all species, with the mean blubber P4 concentration for pregnant animals 164 times higher than that of non-pregnant animals. There was no overlap in concentration levels between sexually immature or non-pregnant sexually mature animals and pregnant animals. No significant differences (F = 0.354, p = 0.559) were found between mature non-pregnant and immature *D* *. capensis* and *P* *dalli* , suggesting P4 level is not indicative of maturity state in female delphinoids. P4 concentrations in relation to reproductive state were remarkably similar across species. All samples were analyzed with two different enzyme immunoassay kits to gauge assay sensitivity to measure progesterone in small samples, such as biopsies. With the technique now validated for these cetacean species, blubber P4 is a reliable diagnostic of pregnancies across multiple species, and thus expands the utility of this method to study reproduction in free-ranging cetaceans using biopsies.

## Introduction

A new field is emerging that utilizes hormones in the blubber of wild cetaceans to study demography; pregnant females have been reliably identified by their blubber progesterone (P4) concentrations in several cetacean species. Determining the proportion of pregnant females in a free-ranging cetacean population can yield highly valuable information in the management of threatened stocks. With this tool we can estimate reproductive rates, project population growth, and compare reproduction with environmental or anthropogenic factors. However, to more broadly implement this method with field-collected samples it is necessary to know the level of variation of blubber P4 expected in pregnant animals of different species. Examining animals of known reproductive status among a suite of species can provide the necessary validation.

Previously, the reproductive endocrinology of small delphinids was predominately studied by quantifying serum progesterone levels in relation to female reproductive state. Correlations between serum progesterone concentration and reproductive status have been examined in fishery caught striped dolphins, *Stenella* *coeruleoalba* [1] and incidentally killed Dall’s porpoises, *Phocoenoides* *dalli* [2,3]. It is also a common practice with captive cetaceans to use serum to monitor hormone levels. For example, Sawyer-Steffan et al. [4] studied the presence of progesterone and estrogens in non-pregnant and pregnant captive Atlantic common bottlenose dolphin, *Tursiops* *truncatus* , in order to implement a successful captive breeding program. In addition, there is limited information on serum progesterone during estrus in Hawaiian spinner dolphins, *Stenella* *longirostris* , in captivity [5]. While this technique is effective when examining dead or captive dolphins, only one study has successfully measured serum progesterone in live wild cetaceans [6]. However, such sampling of wild animals is rare and not feasible for most populations. Also, repeat sampling over time is required to confirm pregnancies [4]. This constraint greatly reduces the utility of serum progesterone when monitoring reproduction in wild populations.

Progesterone levels in biological samples other than blood have been successfully measured and used to assess pregnancy state. For example, progesterone and its metabolites have been shown to vary with pregnancy, lactation, and sexual maturity state in fecal samples from right whales, *Eubalaena* *glacialis* [7]. Progesterone and its metabolites quantified from urine also correlate with reproductive condition, but urine samples can only be obtained from captive animals where trainers are able to routinely and safely collect them [8]. Milk progesterone concentration was indicative of reproductive state in captive *Tursiops* *truncatus* [9]. Yoshioka et al. [10] quantified progesterone from muscle samples of pregnant and immature minke whales, *Balaenapterabonarensis* , to examine relative differences in hormone concentration. Although the authors found significant differences between these reproductive groups, muscle is not a practical tissue to sample from live free ranging individuals. Finally, hormone quantification from whale blow samples has been attempted, but the results are not yet sufficiently consistent to be used reliably [11].

A promising, alternative approach is to quantify progesterone found in blubber. Mansour et al. [12] first examined pregnancy in harvested *Balaenaptera* *acutorostrata* and concluded that blubber progesterone was a viable option to assess pregnancy state. They speculated that it had potential utility in free-ranging whales. Subsequently, Kellar et al. [13] successfully demonstrated the method for several small delphinid species: short-beaked common dolphin ( *Delphinus* *delphis* ), northern right whale dolphin ( *Lissodelphis* *borealis* ), and Pacific white-sided dolphin ( *Lagenorhynchus* *obliquidens* ). According to this study blubber progesterone concentration was a reliable indicator of pregnancy but was unable to distinguish between different stages of pregnancy (i.e. early or late). Perez et al. [14] confirmed the efficacy of this tool in *Tursiops* *truncatus* and long-finned pilot whales ( *Globicephala* *melas* ). These studies indicate that blubber may be more useful than blood serum for detecting pregnant female cetaceans because blubber retains more progesterone per gram of tissue than serum, appears to be less variable, and does not require recurrent sampling [12]. Blubber samples are relatively easy to obtain from wild cetaceans using standard biopsy darts and thus provide a means of applying the methodology to wild populations of cetaceans.

The purpose of this study was to quantify and compare concentrations of progesterone in the blubber of four additional species of small delphinid: the long-beaked common dolphin ( *Delphinus* *capensis* ), the pantropical spotted dolphin ( *Stenella* *attenuata* ), the eastern spinner dolphin ( *S* *. longirostris* ), and one porpoise, the Dall’s porpoise ( *Phocoenoides* *dalli* ). The blubber samples were obtained from fishery-bycatch and stranded specimens for which maturity status had been assessed via inspection of the reproductive tract. The intent here was to examine the correlation of blubber progesterone level with reproductive status, maturity state, and species.

## Materials and Methods

### Ethics Statement

All samples for this study were collected postmortem after carcasses were recovered by the National Marine Fisheries Service (NMFS) Southwest Region Marine Mammal Stranding Network or the eastern tropical Pacific purse-seine and California/Oregon Gill net Observer Programs. No animals were directly targeted and killed for this or any other study. Specimens collected by both of the observer programs were incidentally killed in fishery nets. Incidental bycatch of non-threatened marine mammals is permitted through the NMFS Marine Mammal Authorization Program under the Marine Mammal Protection Act (16 U.S.C. 1371(a)(5)). Response to and sample collection from dead, stranded animals by NMFS is covered under the MMPA (16 U.S.C. 1421). Given that samples were taken from these incidental mortalities no IACUC review was needed.

### Samples

The blubber samples in this study were collected postmortem from female dolphins between 1992 and 2005 from the eastern tropical Pacific purse-seine tuna fishery, the California/Oregon gill net fishery, and strandings along the San Diego coastline. Collection protocols were the same in the two fishery observer programs and the stranding program [15]. Kellar et al. [13] found no variation in progesterone by sampling location so blubber samples in this study were taken from the dorsal mid-thoracic area, which is targeted when sampling via remote biopsy. The sample set included 18 *D* *. capensis* , eight *S* *. attenuata* , six *S* *. longirostris* , and eight *P* *. dalli* (See Table 1 for sample sizes relative to reproductive status). Of these, 10 *D* *. capensis* and one *P* *. dalli* were stranded animals and the remainder of the samples represented fisheries bycatch. Pregnancy was determined by the presence or absence of a fetus. Although an embryo in the very early stage of pregnancy might not be detected by a visual inspection, it is unlikely that this occurred in this study as all specimens with a corpus luteum had a corresponding fetus. An animal was determined to be sexually mature if one or more corpora were present in the ovaries. In cases in which corpus count was not conducted (e.g., no ovaries were collected) we utilized the ratio of total length of the specimen relative to mean total length at sexual maturity for that species as a proxy indicator of maturity status for that individual. This included 10 immature and six mature specimens. The length at sexual maturity (LSM) that we used for *D* *. capensis* , *P* *. dalli* , *S.* attenuata, and *S* *. longirostris* was 197 cm, 181 cm, 166 cm, and 171.2, respectively [16,17,18,19]. In the absence of a direct measurement of LSM for *D* *. capensis* we calculated LSM in this species as 95% of the mean length of adult female *D* *. capensis* as was reported in Heyning and Perrin [17,18]. All of the samples were stored at -20^°^C until the time of extraction.

**Table 1:** A summary of the mean progesterone level (P4) in ng/g for all four species in different reproductive states.

	**Immature**		**Mature Non-Pregnant**		**Mature Pregnant**	
	**Mean P4 (ng/g)**	**n**	**Mean P4 (ng/g)**	**n**	**Mean P4 (ng/g)**	**N**
** *Delphinus* *capensis* **	3.19	8	3.67	8	152.75	2
** *Stenella* *attenuata* **	1.03	5	1.05	1	435.08	2
** *Stenella* *longirostris* **	0.65	3	0.58	1	596.19	2
** *Phocoenoides* *dalli* **	2.31	3	8.29	4	1250.22	1
***AllSpecies***	2.08	19	4.58	14	516.9	7

### Extraction

The hormone extractions were carried out in triplicate following the methods of Kellar et al. [13] with a few modifications to increase the efficiency of the extraction. Samples weighing approximately 0.15 g were homogenized nine times at a speed of five m/s for 45 second intervals. Five hundred µL of the homogenate was then transferred to a 12 x 100 mm borosilicate culture tube and combined with 2 mL of 4:1 ethanol acetone. The samples were vortexed using a multitube vortex and then centrifuged at 4000 rpm for 15 min. The supernatant was transferred into new glass tubes and evaporated. Next, 2 mL of diethyl ether was added to the tubes, the samples were vortexed and centrifuged again, and the supernatant was collected and evaporated. The residue was resuspended in 1000 µL of acetonitrile, vortexed, and 1000 µL of hexane was added to the mixture. After the solution was vortexed and centrifuged again, the acetonitrile layer was aspirated into a new tube and the process was repeated with another 1000 µL of hexane. The final portion of acetonitrile was collected and evaporated. The remaining residue was centrifuged at 4000 rpm for five minutes and stored at -20^°^C.

### Progesterone Enzyme Immunoassay

To prepare the samples for the enzyme immunoassay (EIA), they were suspended in varying amounts of phosphate-buffered saline (PBS, pH 7.5 with 1% bovine γ-globulin). In order to bring the samples to an accurate detection range on the EIA, non-pregnant individuals were resuspended in either 100 µL or 200 µL and those that were pregnant in 1000 µL. All samples were first reconstituted in 100 µL of PBS and then vortexed in the multitube vortex for 15 minutes. We used an EIA kit 900-011 (Enzo Life Sciences, Farmingdale, NY; formerly Assay Designs) that has 100% reactivity with P4 and 5α-Pregnane-3,20-dione (a related synthetic immunoreactive progestin) in each sample. The assay detection limits were between 15 and 500 pg/ml. Therefore, samples that exceeded this range had to be diluted further to be accurately measured. These samples were diluted at 1:100, 1:20, 1:5, 1:3, and 1:2 depending on their original EIA measurements such that the final measurements would fall within the range of the control samples. The intra-assay coefficient of variation (CV) was between 4.9% and 7.6% and the inter-assay CV between 2.7% and 6.8%. Each sample was individually vortexed prior to quantifying P4 concentration.

We determined the extraction efficiency using spiked samples according to Kellar et al. [13]. These extraction control samples were spiked with 0 ng, 10 ng, and 30 ng of P4. The percentage of P4 that was recovered after extraction was calculated and each assay value was adjusted to the standard prior to analyses

### Assay Evaluation

Kellar et al. [13] utilized an EIA kit designed by Diagnostic Systems Laboratory (DSL-10-3900; Webster, TX) for data analysis, but we found the kit provided by Enzo Life Sciences (formerly Assay Designs heretofore referred to as AD) to be more sensitive and precise after testing a subset of our samples using both kits. To confirm this, samples from 40 different individuals were tested with both EIA kits; these were processed identically irrespective of the measurement assay.

### Data Analyses

To evaluate the variation in blubber P4 concentration between species and with pregnancy state we first calculated the mean blubber P4 concentration for each sample. Immature animals of all species were compared via a one-way ANOVA. A two-way ANOVA was used to compare immature and mature non-pregnant *D* *. capensis* and *P* *. dalli* , the two species that had sufficient sample size for such a comparison. Given non-significant results from the two ANOVAs it was assumed that mature non-pregnant *S* *. attenuata* and *S* *. longirostris* (both with an n=1) would not differ significantly from this pattern and the data were pooled for further comparisons. To examine how robust this technique is at diagnosing pregnancy regardless of species, all data were pooled by reproductive class and compared by using student’s t-tests. All non-pregnant specimens were also pooled and compared with all pregnant specimens.

### Assay evaluation

Paired t-tests were performed to compare the results obtained by samples replicated using both AD and DSL EIA kits. First, data from all samples were pooled for analysis and compared by kit type. Next, the samples were split by category (immature, mature non-pregnant, and pregnant) and re-evaluated for differences associated with kit type.

## Results

### P4 concentration comparisons

**Figure 1:**
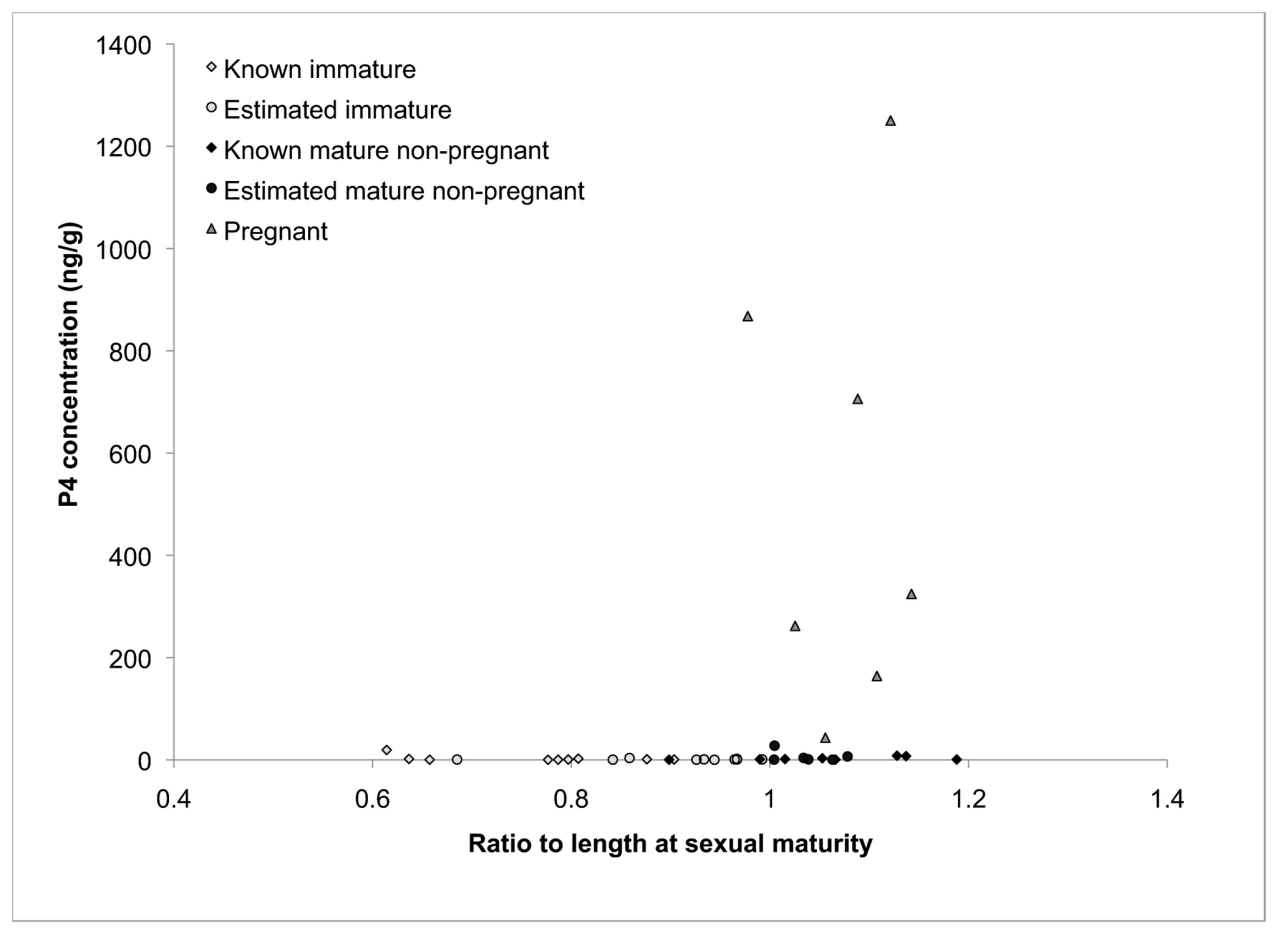
The P4 concentration for each sample in the data set is plotted by state of sexual maturity and pregnancy. The animals are categorized along the x-axis according to their ratio to length at sexual maturity (i.e. the length of the animal relative to the average length at which other animals of the same species reach sexual maturity).

**Figure 2:**
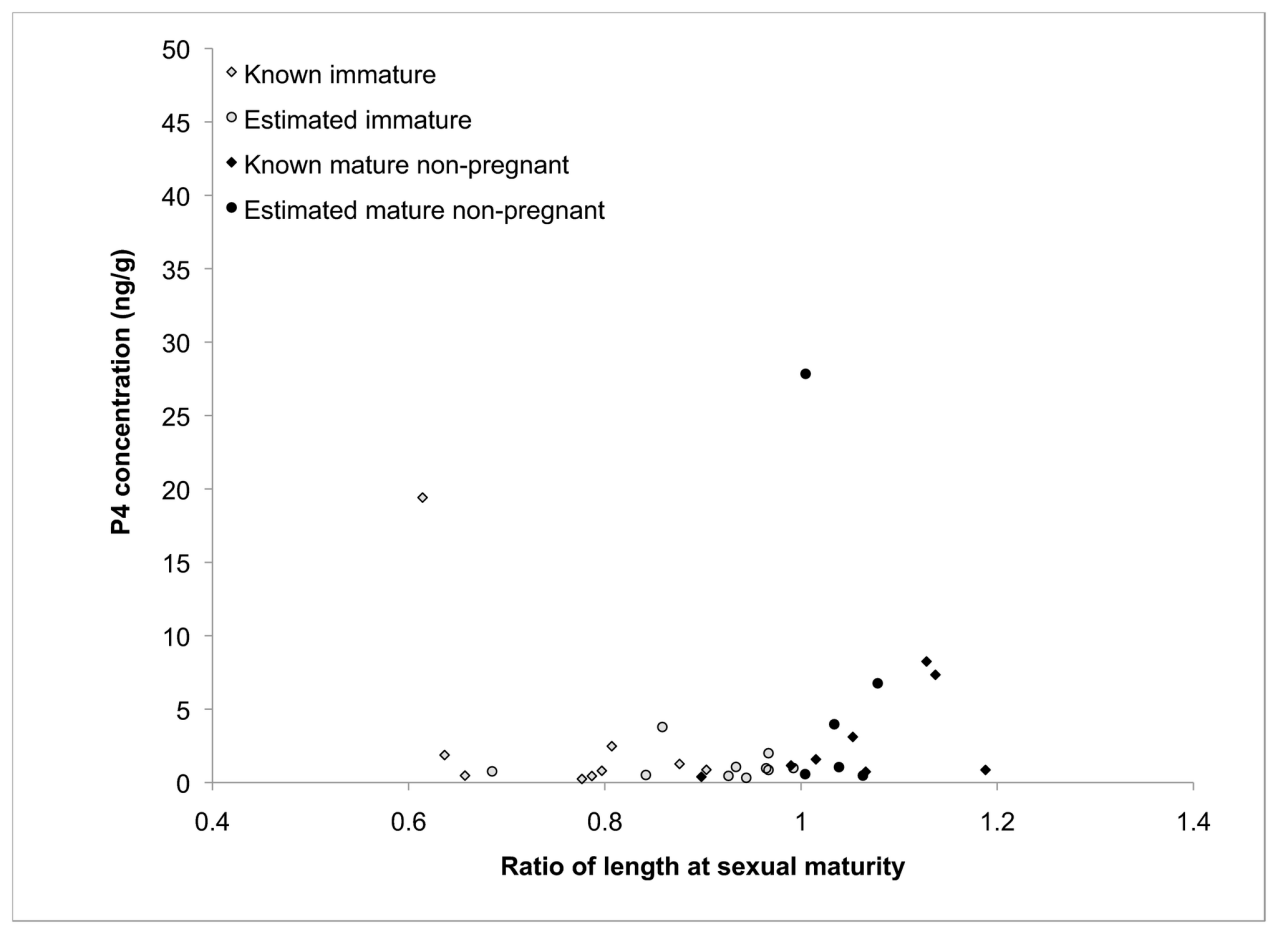
Non-pregnant individuals are plotted with their corresponding P4 concentrations. Animal length is standardized for the plot as the ratio of total body length to the species-specific average length at sexual maturity.

No differences were observed when comparing immature animals of different species (F = 0.351, *p* = 0.789). Similarly, there was no statistical significance found between species or maturity state of non-pregnant *D* *. capensis* and *P* *. dalli* (F = 0.354, *p* = 0.559 and F=1.056, *p* = 0.317 respectively). Pregnant animals had higher P4 concentrations than non-pregnant individuals irrespective of species (t = -7.1, *p* = 1.79E-08; Table 1 and Figure 1). When pooled, the mean blubber P4 concentration in pregnant animals (516.90 ng/g) was 246 times higher than that measured in immature (2.08 ng/g) animals, and 112 times higher than the mean of mature non-pregnant (4.58 ng/g) individuals. One sample from a pregnant *Delphinus* *capensis* contained a value considerably lower than expected (43.47 ng/g). However, this value is still well above the mean P4 concentration in all non-pregnant animals, which is approximately 3.14 ng/g. All other pregnant individuals had concentrations ranging from 164 ng/g to 1250 ng/g (Figure 1). The majority of non-pregnant individuals displayed P4 concentrations below 10 ng/g (Figure 2). Only two individuals had mean P4 levels higher than 10ng/g (19.41 ng/g and 27.84 ng/g), but even these were below the lowest concentrations observed in pregnant animals.

**Figure 3:**
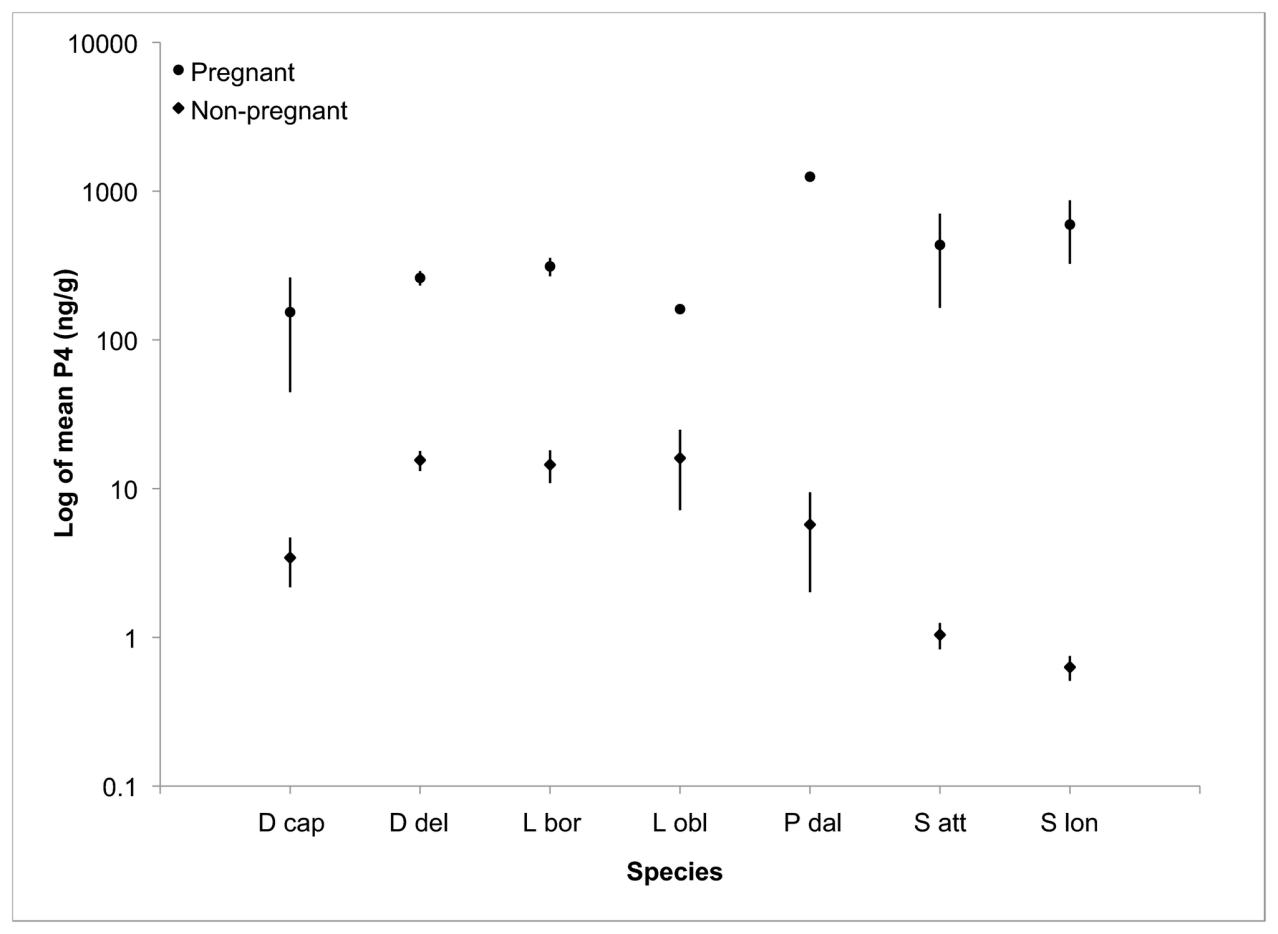
The mean P4 values for this study are combined with those from Kellar et al [13] to illustrate species-specific variability. Note that the y-axis is presented on a log_10_ scale to show a wide range of P4 values; whiskers indicate the standard error of the non-log values (D cap – *Delphinus* *capensis* , D del - *Delphinus* *delphis* , L bor - *Lissodelphis* *borealis* , L obl - *Lagenorhynchus* *obliquidens* , P dal - *Phocoenoides* *dalli* , S att - *Stenella* *attenuata* , S lon - *Stenella* *longirostris* ).

**Figure 4:**
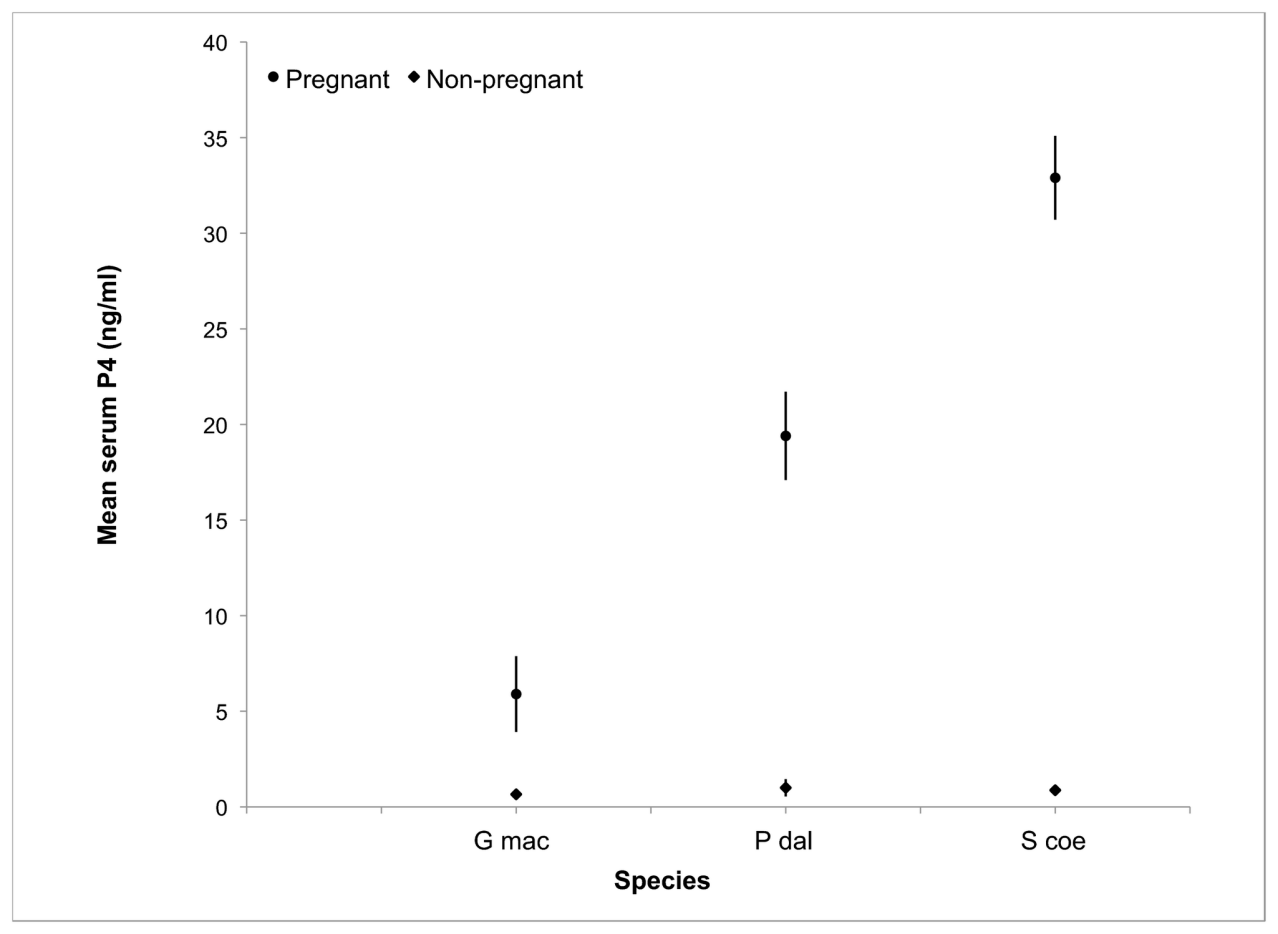
The P4 concentration in blood serum of pregnant and non-pregnant animals. The data were taken from previous studies on *Phocoenoides* *dalli* (P dal [2,3]), *Stenella* *coeruleoalba* (S coe [1]), and *Globicephala* *macrorhynchus* (G mac [1]).

When combined with data from Kellar et al. [13], blubber P4 concentrations from all seven species successfully distinguish pregnancy states (Figure 3). The difference between pregnant and non-pregnant blubber P4 was much greater than that between pregnancy states in serum P4 (Figure 4). Sample size in this study was limited to one or two for some classifications and do not allow a complete statistical analysis for certain comparisons. While performing statistical analyses on the small sample size available for this study was not ideal, waiting to obtain additional samples would take a considerable amount of time. As a burgeoning field, there is a current need for baseline data on blubber hormone levels across a variety of species and the minimal sample size should not detract from the value of the data provided here.

### Assay Evaluation

**Figure 5:**
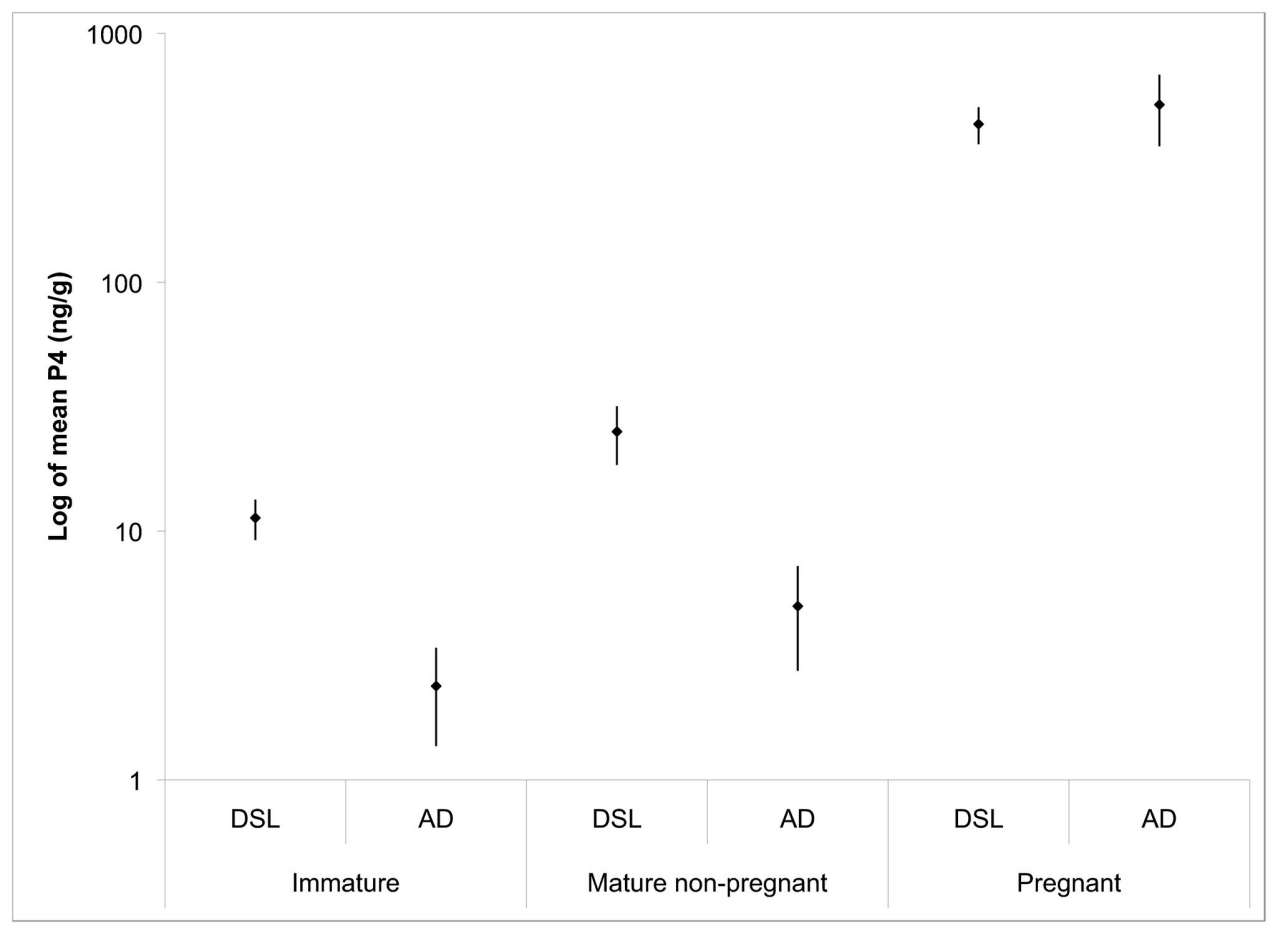
The mean P4 values obtained from Diagnostic Systems Laboratory (DSL) and Enzo Life Sciences/Assay Designs (AD) EIA kits according to reproductive class. Note that the y-axis is presented on a log_10_ scale to show a wide range of P4 values; whiskers indicate the standard error of the non-log values.

No difference was present between the blubber P4 concentrations measured with the AD EIA kit and those measured from the DSL kit when all reproductive classes were combined (Table 2, t = 0.087, *p* = 0.93). However, after the data were split according to reproductive class, the blubber P4 level for both immature (t = -3.89, *p* = 0.00042) and mature non-pregnant (t = -2.77, *p* = 0.011) groups differed between the two kits (Figure 5). Immature and mature non-pregnant samples analyzed with DSL kits (mean = 11.29 ng/g and 25.077 ng/g respectively) had higher blubber P4 concentrations than those run with AD kits (mean = 2.38 ng/g and 4.99 ng/g respectively). In both immature and mature non-pregnant groups, samples processed with the DSL kit contained a higher standard error in blubber P4 concentration than those with the AD kit (immature: AD = 1.01, DSL = 2.09 and mature non-pregnant: AD = 2.25, DSL = 6.64). There was no difference between pregnant specimens examined with both kits but the standard error was higher when using the AD kit (165.28) versus the DSL kit (73.37). The AD assay provided more precise values because the DSL assay is less sensitive at measuring progesterone between a range of 0.3 and 80 ng/ml, whereas the AD assay detects values between 15 to 500 pg/ml. Because AD kits can more accurately quantify lower levels of P4 than DSL kits, it is more suitable for use with biopsy samples, which often contain small amounts of blubber and increased sensitivity of the assay is critical for measuring P4. Therefore, we chose to use only the results obtained from AD kits for our analysis in this paper.

**Table 2:** The p-values for the comparison between Diagnostic Systems Laboratory (DSL) and Enzo Life Sciences/Assay Designs (AD) progesterone enzyme immunoassay kits.

	**Total**		**Immature**		**Mature Non-pregnant**		**Mature Pregnant**	
	**Mean**	**SE**	**Mean**	**SE**	**Mean**	**SE**	**Mean**	**SE**
**DSL**	93.44	29.42	11.29	2.09	25.08	6.64	431.63	73.37
**AD**	97.98	43.45	2.38	1.01	4.99	2.25	516.9	165.28
***P***	0.93		**0.00042**		**0.011**		0.65	

## Discussion

This study adds baseline data and validation for blubber P4 concentrations of four additional cetacean species, including one porpoise, to the existing research on blubber hormones. While we analyzed a relatively small sample set, the results contribute knowledge about similarities and differences among cetacean species. The most important similarity observed for the four cetacean species in this study was that, despite minor fluctuations, progesterone concentrations were diagnostic of pregnancy.

We found that pregnant individuals had significantly higher blubber P4 levels compared to immature and mature non-pregnant individuals, regardless of species. Only one pregnant individual had a relatively low P4 level when compared to other pregnant individuals. This specimen, from a beach stranding, was highly decomposed, which could explain some loss of blubber P4. This specimen was classified as a code 4 according to Geraci and Lounsbury [20]. Stranding state is on a scale from 1–5, 1 being a live animal and 5 being mummified or skeletal remains. A code 4 is characterized as portraying advanced decomposition [20]. To our knowledge, this is the first evidence of P4 loss due to poor sample quality or post-mortem degradation, and additional work should be conducted to examine the effect of decomposition on P4 levels in blubber. Kellar et al. [13] tested for progesterone loss through decomposition by exposing blubber samples to the elements for 52 hours and found no significant change. However, these samples were composed only of the excised blubber and cannot account for any internal decomposition processes that may occur within a whole specimen. If there is a relationship between hormone concentration and level of decomposition, it may be possible to estimate the rate of loss and correct for the degradation. Still, the individual in this study maintained a significantly higher P4 concentration than all other non-pregnant cetaceans. Even including this one anomalous sample, blubber P4 was an accurate indicator of pregnancy in all species examined.

While the majority of non-pregnant samples displayed very low blubber P4 levels, two samples in this study, one mature non-pregnant *Phocoenoides* *dalli* and an immature *Delphinus* *capensis* , showed near-ambiguous P4 levels (27.84 ng/g and 19.41 ng/g respectively). A possible reason for higher blubber P4 levels in the mature non-pregnant *Phocoenoides* *dalli* may be due to calving interval. This individual happened to be accompanied by a neonate that, according to its size, was likely under 1 month old. The high P4 level found in the mother is likely the result of the recent birth of her calf [16]. Kellar et al. [13] tested the blubber P4 level in a post-pregnant *Lissodelphis* *borealis* containing a distended uterus. The individual had a mid-range P4 level that was higher than other non-pregnant individuals but 30% lower than pregnant individuals. Because *Phocoenoides* *dalli* reproduces annually [16], mature non-pregnant individuals are disproportionately more likely to have recently given birth at the time of sampling and would be expected to have higher mean residual blubber P4. However, because our study had such a small sample size, a more in-depth analysis is necessary before any conclusion can be made.

The immature *Delphinus* *capensis* with high P4 levels was a stranded year-old calf with severe deformities and an extremely thin blubber layer. The high blubber P4 levels may be attributed to the reduced blubber layer and limited cross reactivity of the P4 EIA with corticosterone, a corticosteroid that can be associated with stress responses in mammals. The AD EIA assay has a cross reactivity with corticosterone of 0.77%. If this specimen was highly stressed due to its health and had a large excess of corticosterone in its system then the P4 EIA would superficially raise the detected P4 level above its actual concentration. Still, this sample does not fall within the ambiguous range and does not interfere with pregnancy diagnosis.

In comparing the blubber P4 level across seven of the delphinoids examined to date, in this study and those in Kellar et al. [13], we see that there is a clear division between pregnant and non-pregnant animals. In these seven species, non-pregnant animals typically do not have a blubber P4 level above 10 ng/g while pregnant animals usually maintain P4 levels well above 100 ng/g. The large magnitude of difference between pregnant and non-pregnant animals suggests that a pregnancy diagnosis can be made on a sample from a species that has not yet been analyzed. To achieve this, any individual with a blubber P4 value below 30 ng/g would be considered non-pregnant and those with values higher than 50 ng/g would be classified as pregnant. Individuals with blubber P4 level between 30 and 50 ng/g P4 would fall into the ambiguous category. Such ambiguous values are to be expected; in early stages of pregnancy blubber P4 values transition from low to high concentrations and soon after parturition P4 concentrations fall from high to low. If serum and milk were to be sampled during this transition we would not expect a high P4 concentration. However, there is some lag time associated with the change in concentration of P4 in the blubber causing a higher occurrence of intermediate values. Possible causes of high progesterone values without pregnancy include ovulation, ovarian cysts, and pseudopregnancy. However, we have no empirical evidence that these occur in high frequency in wild delphinoids. For the seven species we have tested, 0 of 150 individuals showed sign of ovulation without conception (corpus luteum present without a fetus). This suggests that the period of time that a female delphinoid is in this state (ovulating without conceiving) is a small fraction of its life and therefore less likely to be sampled. In total we have had four ambiguous measurements (30-50ng/g) out of the 150 animals of known reproductive condition. This represents 2.7% of the sample set and includes the highly degraded pregnant animal ( *Phocoenoides* *dalli* ), the individual in Kellar et al. [13] with a distended uterus ( *Lissodelphis* *borealis* that was pregnant just prior to sampling), and two other specimens, each of a different species ( *Delphinus* *delphis* and *Lagenorhynchus* *obliquidens* ) whose ambiguous values have no apparent explanation.

Blubber P4 promises to be a much more consistent diagnostic of pregnancy in wild delphinoids than serum P4. The degree of difference in P4 content in the blubber of pregnant versus non-pregnant delphinoids is much greater than that seen in blood serum studies. In pregnant cetaceans, the serum P4 levels do not reach the same high levels as seen in blubber and are likely more variable. This is perhaps because P4 is secreted into the bloodstream in pulses, which causes larger fluctuations in P4 concentration within the circulatory system [21]. Conversely, it is thought that blubber accumulates (and conversely disperses) P4 continuously as the level in the blood rises and falls regularly [13]. With highly fluctuating concentrations of P4 in the serum it is difficult to diagnose a pregnancy without monitoring it over a period of time [4]. Additionally, it is our experience that we can obtain reliable blubber P4 measurements from samples weighing 50mg or more, making this technique feasible for use in biopsies obtained from free-ranging animals.

## Conclusions

Despite minor variations of blubber P4 concentration with respect to different assays, all pregnant specimens had a substantially (and significantly) elevated level of P4 when compared to non-pregnant animals. The mean blubber P4 level in pregnant individuals was 164 times that found in non-pregnant individuals. Our results show that blubber P4 is an effective pregnancy diagnostic with a high degree of certainty from a fairly small amount of blubber and we think it is a useful technique ready to be applied to biopsy samples collected in the field from various cetaceans.
